# The effects of different accumulated pressure-time integral stimuli on plantar blood flow in people with diabetes mellitus

**DOI:** 10.1186/s12891-021-04437-9

**Published:** 2021-06-18

**Authors:** Yijie Duan, Weiyan Ren, Liqiang Xu, Wenqiang Ye, Yih-Kuen Jan, Fang Pu

**Affiliations:** 1 Advanced Innovation Center for Biomedical Engineering, School of Biological Science and Medical Engineering, Beihang University, Beijing, People’s Republic of China; 2 Key Laboratory of Human Motion Analysis and Rehabilitation Technology of the Ministry of Civil Affairs, National Research Center for Rehabilitation Technical Aids, Beijing, People’s Republic of China; 3 Rehabilitation Engineering Laboratory, Department of Kinesiology and Community Health, University of Illinois at Urbana-Champaign, Champaign, IL USA

**Keywords:** Diabetic foot ulcers, Plantar skin blood flow, Accumulated pressure-time integral, Weight-bearing exercise, Microcirculation

## Abstract

**Background:**

Exercise, especially weight-bearing exercise (e.g. walking), may affect plantar tissue viability due to prolonged repetitive high vertical and high shear pressure stimulus on the plantar tissue, and further induce development of diabetic foot ulcers (DFUs). This study aimed to investigate the effects of different accumulated pressure-time integral (APTI) stimuli induced by walking on plantar skin blood flow (SBF) responses in people with diabetes mellitus (DM).

**Methods:**

A repeated measures design was used in this study. Two walking protocols (low APTI (73,000 kPa·s) and high APTI (73,000 × 1.5 kPa·s)) were randomly assigned to ten people with DM and twenty people without DM. The ratio of SBF measured by laser Doppler flowmetry after walking to that before (normalized SBF) was used to express the SBF responses.

**Results:**

After low APTI, plantar SBF of people with DM showed a similar response to people without DM (*P* = 0.91). However, after high APTI, people with DM had a significantly lower plantar SBF compared to people without DM (*P* < 0.05). In people with DM, plantar SBF in the first 2 min after both APTI stimuli significantly decreased compared to plantar SBF before walking (*P* < 0.05).

**Conclusions:**

People with DM had a normal SBF response after low APTI walking but had an impaired SBF response after high APTI walking, which suggests that they should avoid weight-bearing physical activity with intensity more than 73,000 kPa·s and should rest for more than 2 min after weight-bearing physical activity to allow a full vasodilatory response to reduce risk of DFUs.

## Background

Diabetic foot ulcers (DFUs) are one of the most serious complications of diabetes mellitus (DM) [1]. The International Diabetes Federation reports that there are approximately 463 million people (20–79 years) with diabetes [2]. The prevalence of DFUs is about 6.3% worldwide [3], and one in five people with DFUs will eventually have lower-extremity amputation [4]. It significantly affects the quality of life of people with DM.

The underlying risk factors for the development of DFUs include peripheral neuropathy, peripheral vascular disease, abnormal plantar pressure, trauma and infection [1, 5, 6]. The impaired microvascular function caused by these factors along or in combination may decrease skin blood flow of diabetic foot tissue under abnormally high external mechanical stress stimulus during daily walking, which will lead to DFUs [5–9]. Therefore, it is important to improve plantar microvascular function for reducing risk of DFUs.

Studies have shown that exercise can improve peripheral microvascular function in people with DM [10–12]. Suntraluck et al. demonstrated that 12 weeks of supervised cycling exercise improved the endothelium-dependent flow-mediated dilation in older people with DM [11]. Liao et al. showed that exercise is beneficial to improve the ankle brachial index [12]. Wu et al. demonstrated that faster walking speeds (i.e. 6 and 9 km/h) could significantly increase plantar skin blood flow within the same duration, and concluded that faster walking speeds should be suggested for people with DM for improving microvascular function [13]. However, Burnfield et al. showed that faster walking speeds increased plantar pressure at the toes, medial metatarsal heads and heel [14]. It indicates that the diabetic plantar tissue may be subjected to a high level of cumulative pressure during the repetitive high stress (faster walking) over the same duration, which has been considered to negatively affect the plantar blood perfusion and plantar tissue viability [5, 15–17]. Therefore, it is crucial to further explore the response of plantar microcirculation to different amounts of cumulative pressure, which will help to further clarify the degree of foot ulcer risk when pursuing various exercise with different accumulated pressure stimuli in people with DM [5, 18].

Pu et al. previously proposed the accumulated pressure-time integral index (APTI, the summation of pressure-time integral over a certain time period), and indicated that the time factor of plantar pressure should be considered [18], because previous studies have found that some people with DM developed DFUs even though they had low peak plantar pressure (PPP) [19, 20]. They demonstrated that the plantar SBF significantly decreased after the same APTI stimulus in people with DM with high PPP and low PPP. They also found that no significant relationship was observed between PPP and blood flow responses of the plantar foot before and after the same APTI stimulus [18]. Therefore, the sole use of PPP may not be sufficient to quantify the risk of DFUs. APTI may have a crucial complementary advantage in quantifying the risk of DFUs associated with various physical activities in people with DM due to its inclusion of both time and pressure factors [5]. However, the previous study [18] only measured the blood flow responses within 2 min after one level of APTI stimuli. Zhu et al. demonstrated that plantar SBF in response to 10-min walking lasted at least 3 min in healthy participants [21]. Due to impaired microvascular function in people with DM [22], it is reasonable to speculate that the recovery time in people with DM would be longer than 3 min observed by Zhu et al. Therefore, the skin blood flow response within 2 min may not be sufficient to fully characterize plantar tissue in response to the APTI stimulus.

Jan et al. demonstrated that people with DM have an impaired plantar microvascular reactivity after local mechanical loading [22]. Petrofsky et al. compared the effects of four transient (30s) local pressure loading (15, 30, 45, and 60 kPa) on the 2nd metatarsal head of young healthy people, older healthy people and people with DM. They found that the post-pressure skin blood flow of young healthy people was significantly higher with the 60 kPa pressure than with other pressure applied, while no statistical difference was observed between skin blood flow after any pressure applied in people with DM [23]. It indicated that people with DM had an impaired microvascular reactivity under the high local pressure stimulus. However, such a transient local pressure stimulus does not reflect the time of regular exercise in our daily activities, and the plantar tissue is usually subjected to repetitive vertical and shear pressure stimuli during daily walking [5, 15, 19]. The impaired microvascular reactivity might deteriorate under repetitive pressure applied over a long period of time, which may cause a local inflammatory response and increase the risk of DFUs [5, 18, 24]. Therefore, it is necessary to investigate the blood flow responses of the diabetic plantar tissue under different levels of APTI stimuli, in order to investigate the safe range of APTI for the plantar tissue in people with DM during weight-bearing exercise.

This study aimed to investigate the effects of different APTI stimuli on plantar blood flow responses in people with diabetes. We hypothesized that people with diabetes have a similar blood flow response after the low APTI stimulus (a safe plantar tissue stress [18]) and have a significantly lower blood flow response after the high APTI stimulus (theoretically high plantar tissue stress [18]) compared to people without diabetes.

## Methods

A repeated measures design was used in this study to analyse the effects of different APTI stimuli induced by walking on plantar skin blood flow responses in people with and without diabetes.

### Participants

People with type 2 diabetes mellitus (DM group) between 60 and 79 years of age and healthy participants without DM (healthy group) between 20 and 29 years of age were recruited from nearby hospitals and universities. Considering the better vascular function in young healthy people, this study recruited young healthy people as the control group to establish the normal response of plantar tissue to various accumulated pressure-time integral stimuli. The exclusion criteria of people with DM included the history of foot ulcers or amputation, the symptoms such as redness, callus, inflammation, intermittent claudication (e.g. unable to walk independently for 30 min [25, 26]), an ankle brachial index (ABI) less than 0.9 or greater than 1.3, and the metabolic diseases such as systemic inflammation, lower limb edema, or malignant tumors. The exclusion criteria of healthy participants without DM included having diabetes, vascular diseases, metabolic diseases, or callus.

All participants were briefed on the study purposes and test methods, and written informed consent was obtained. This study was conducted in accordance with the Declaration of Helsinki. Institutional review boards of all participating centers approved the study.

### Equipment

Skin blood flow (SBF) was noninvasively measured using Laser Doppler flowmetry (LDF) (PeriFlux 5000, 457, Perimed, Stockholm, Sweden). This device delivered a low-power beam (2 mW) of helium-neon light (780 nm) transmitted through fibre optics into the tissue and the light scattered by the tissue is collected by a probe photodetector. In order to ensure the reliability of data collection, the LDF probe was calibrated before each measurement, and each participant was required to enter the laboratory at least 30 min before blood flow measurements to adapt to the laboratory environment. In addition, the SBF data at each stage was continuously monitored for 10 min at a 32 Hz sampling rate of LDF signal, and the probe was attached to the skin surface of the plantar first metatarsal area with adhesive tapes to limit movement artefacts during the measurement.

The custom-made pressure monitoring shoes described in the previous study [18] was used to measure and calculate APTI in real time during walking. This is a feedback system based on real-time plantar pressure measurements. A pressure sensor (FSR-400, Interlink Electronics, Amarillo, United States) is placed under the center of the first metatarsal region on the insole made by Ethylene Vinyl Acetate (EVA, ROKAB, Canada) to monitor real-time pressure during walking. Insoles of different sizes according to people’s foot sizes were customized, and then placed in the matching size of sports shoes of DECATHLON brand. Smartphone was used to receive real-time pressure signal via Bluetooth and would send out an audible alarm when the calculated APTI value of the region of interest of each participant reached the preset threshold (i.e. 73,000 kPa·s and 73,000 × 1.5 kPa·s).

A durometer (model 1600, type OO, Rex Gauge CO., Buffalo Grove, Illinois, USA) was used to measure the soft tissue stiffness of the plantar first metatarsal head in people with DM to avoid the influence of tissue stiffness on skin blood flow response. Because Chao et al. reported an increasement in stiffness of plantar soft tissue in people with DM [27], which may affect the skin blood flow response [22, 28]. This type of durometer shows the relative stiffness of soft tissue by applying an indentation load against the skin surface and expresses the soft stiffness in degrees of Shore (unit: °shore). A higher Shore value indicates a stiffer tissue. During the measurement, the durometer was pressed perpendicularly to the skin surface and the stiffness results were read for five times, and the mean value of five results was calculated for each participant [29].

In this study, the blood glucose level and ABI of people with DM were recorded from the medical records. 10 g Semmes-Weinstein monofilament was used to evaluate whether participants had sensory neuropathy. Firstly, the monofilament was applied on the patient’s hands to demonstrate what the sensation feels like. Then, it was applied perpendicular to the skin surface of four areas of foot (1st, 3rd, and 5th metatarsal heads and distal hallux). The measurements at the same site were repeated three times with at least one ‘mock’ application in which no filament is applied [30]. It was considered normal large-fiber nerve function if the patient could feel the touch of the monofilament at all four areas.

### Experimental procedures

The experiment consisted of two protocols. (1) The low APTI protocol referred to the APTI stimulus at 73,000 kPa·s (low APTI stimulus) [18], which equals to walking for 15 min at 54 steps per minute [31] for a pressure-time integral (PTI) of 90 kPa·s [32]. (2) The high APTI protocol referred to the APTI stimulus at 73,000 × 1.5 kPa·s (high APTI stimulus), which is 1.5 times of the low APTI protocol. The high APTI protocol tested in this study would not cause people with DM to walk at the preferred speed for more than 30 min. The reason to limit to not more than 30-min walking was that studies showed more than half of adults engage in moderate intensity exercise for less than 30 min a day in the United States, the United Kingdom, and Asian countries [33]. Studies have shown that the first metatarsal head is one of the most common areas for foot ulcers [34]; thus, we focused on skin blood flow at the first metatarsal of each participant’s right foot before and after walking.

All research participants performed these two APTI protocols in a random order. Firstly, each participant was asked to rest for 30 min in the laboratory. Then, the plantar SBF over the first metatarsal head was measured for 10 min in the supine position to record the basic blood flow. During this time, the participants kept their bodies still as much as possible and avoided talking to keep the blood flow data stable. After that, they were asked to wear the custom-made pressure monitoring shoes and walked on the treadmill at a speed of 3.2 km/h [35]. When the APTI value reached the preset threshold (73,000 kPa·s or 73,000 × 1.5 kPa·s), they were immediately asked to stop walking and continue to measure plantar SBF for 15 min. After a 30-min washout period, the participants performed the other experiment protocol. The plantar SBF was similarly measured for 10 min, then, the participants still walked on the treadmill at the same speed until the APTI value reached the threshold and have another 15-min measurement of plantar SBF. All tests were conducted in a temperature-controlled room at 24 °C.

### Statistical analysis

In this study, the required sample size was calculated using Power Analysis and Sample Size (PASS 11) software set for paired *t* test. The mean and standard deviation (SD) of paired difference between before and after APTI stimuli in people with diabetes were assumed to be equivalent to that from a prior study (14.35% ± 10.90%) [18]. A minimum of 7 participants was needed for the power of 80% at an alpha level of 0.05.

The mean values of SBF during the baseline period and the recovery period within 14 min were calculated. Normalized SBF was the ratio of the SBF during the recovery period to the SBF during the baseline period to characterize SBF responses. The mean value of SBF per 2 min in the 14-min recovery period was calculated to explore changes in plantar SBF. The first 2-min SBF response was used to compare the results of Pu et al.’s study [18]. In order to preliminarily observe the effect of different tissue stiffness on blood flow response, people with DM were divided into the high tissue stiffness group and the low tissue stiffness group according to the mean value of plantar tissue stiffness of all participants with diabetes measured in this study.

Independent *t* test or Mann-Whitney U test (based on Shapiro-Wilk’s normality test) was used to compare the physiological characteristics between healthy group and DM group, the normalized SBF differences between healthy group and DM group under the same APTI stimulus, as well as the normalized SBF differences between the high tissue stiffness group and the low tissue stiffness group in people with DM. Paired *t* test or Wilcoxon matched-pair signed-rank test (based on Shapiro-Wilk’s normality test) was applied to compare the differences of SBF between the recovery and baseline periods in the two protocols of each group, as well as the differences of normalized SBF between the low APTI stimulus and the high APTI stimulus. All data were represented as mean ± SD, the significance level was set at 0.05. All statistical analyses were performed by SPSS (Version 26.0, IBM, Armonk, NY, USA).

## Results

Ten people with type 2 diabetes mellitus (DM group: Age: 68.1 ± 4.61 years; Body mass index (BMI): 24.76 ± 2.61 kg/m^2^) and 20 healthy participants without DM (healthy group: Age: 24.65 ± 1.04 years; BMI: 21.2 ± 1.71 kg/m^2^) were enrolled in this study. None of people with diabetes was diagnosed with diabetic peripheral neuropathy or foot deformity. The duration of diabetes, fasting glucose, glycated haemoglobin and ABI of people with DM obtained by the medical history, and other demographic information of all participants measured in this experiment are shown in Table 1. No significant differences were observed between two groups in systolic blood pressure (SBP), diastolic blood pressure (DBP) and heart rate. One of the participants with DM only completed the low APTI protocol but not the high APTI protocol due to personal reasons; thus, only the data of the low APTI protocol was included for analysis.

**Table 1:** Demographic and physiological characteristics of participants (Mean ± SD)

	DM group	Healthy group
SBP (mmHg)	127.7 ± 19.06	116.85 ± 8.57
DBP (mmHg)	69.3 ± 11.14	67.75 ± 5.78
Heart rate (bpm)	67.6 ± 9.54	72.15 ± 7.07
Duration of diabetes (years)	12.2 ± 4.52	/
Fasting glucose (mmol/L)	7.16 ± 1.19	/
Glycated haemoglobin (%)	7.2 ± 1.3	/
ABI (right leg)	1.08 ± 0.09	/

*SBP* systolic blood pressure, *DBP* diastolic blood pressure, *ABI* ankle brachial index. No significant differences were observed between two groups in SBP, DBP and heart rate. *SD* standard deviation.

**Fig. 1:**
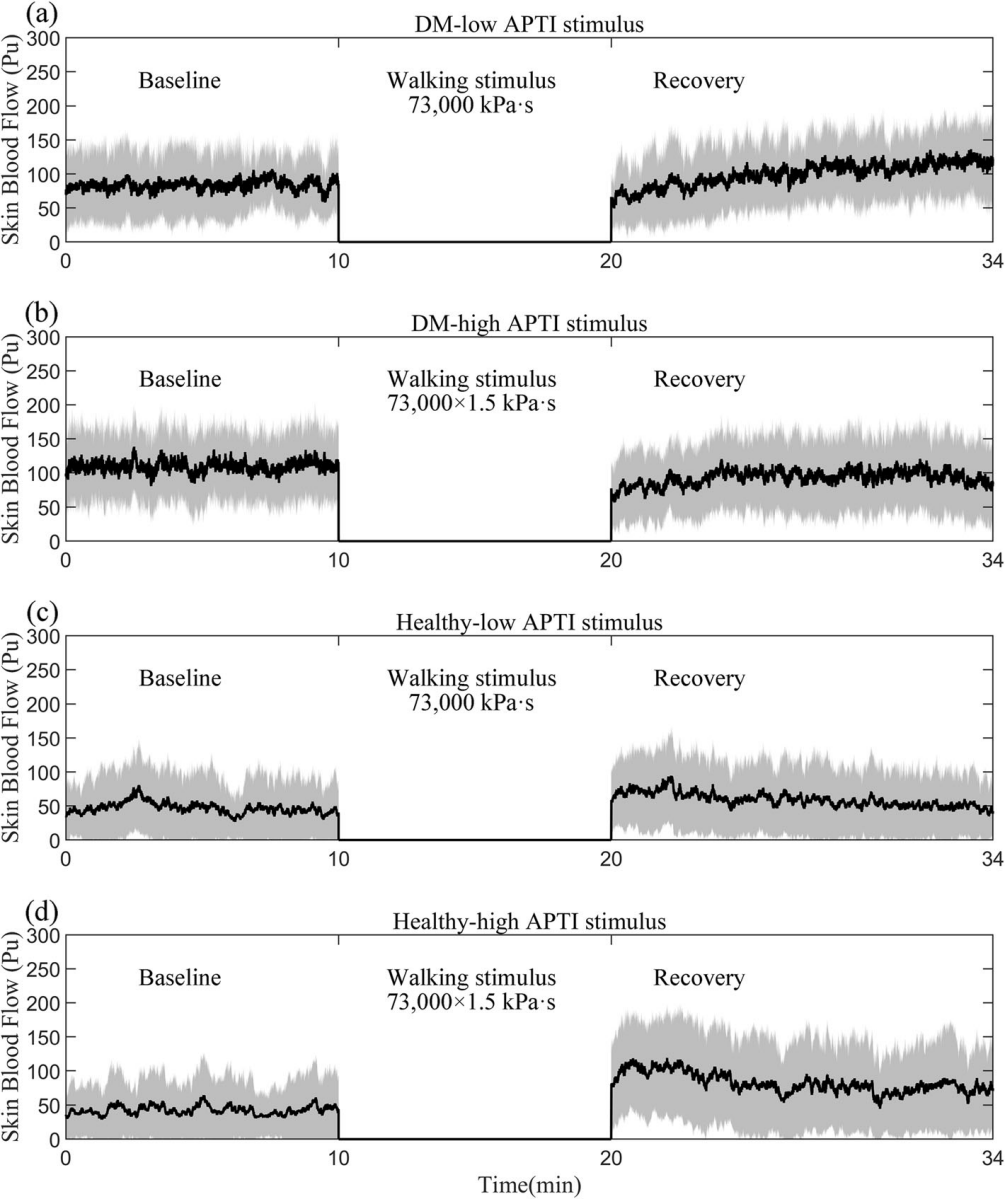
Illustration of plantar skin blood flow before and after different APTI stimuli in people with DM and healthy participants without DM (mean and standard deviation clouds, the black line is mean and the shaded area is standard deviation; Unit: Perfusion unit (Pu)). **a** People with DM after the low APTI stimulus; **b** People with DM after the high APTI stimulus; **c** Healthy participants after the low APTI stimulus; **d** Healthy participants after the high APTI stimulus

**Fig. 2:**
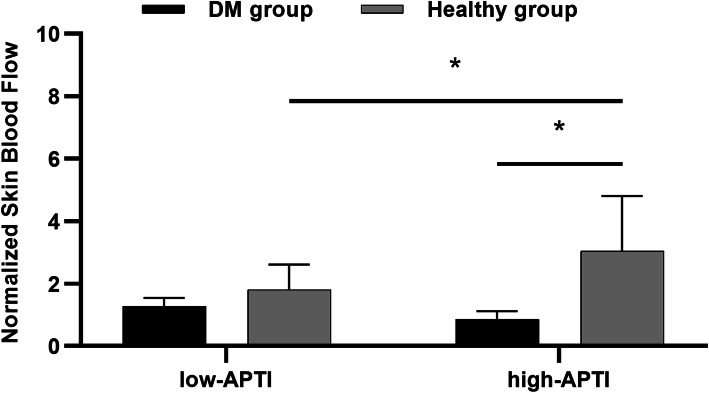
Results of normalized skin blood flow between the high and low APTI stimuli in each group (mean with 95%CI). The symbol “*” indicates a significant difference between each group (*P* < 0.05). Normalized skin blood flow means the ratio of skin blood flow after walking to that before walking

The skin blood flow responses to different APTI stimuli induced by walking from participants in each group were shown in Fig. 1. Normalized SBF of healthy participants (3.05 ± 3.74) was significantly higher than that of people with DM (0.86 ± 0.33, *P* < 0.05) in the high APTI stimulus. Besides, normalized SBF of high APTI stimulus (3.05 ± 3.74) was significantly higher than that of low APTI stimulus (1.81 ± 1.71, *P* < 0.05) in healthy participants. There was no significant difference in the others (*P* > 0.05; Fig. 2).

**Fig. 3:**
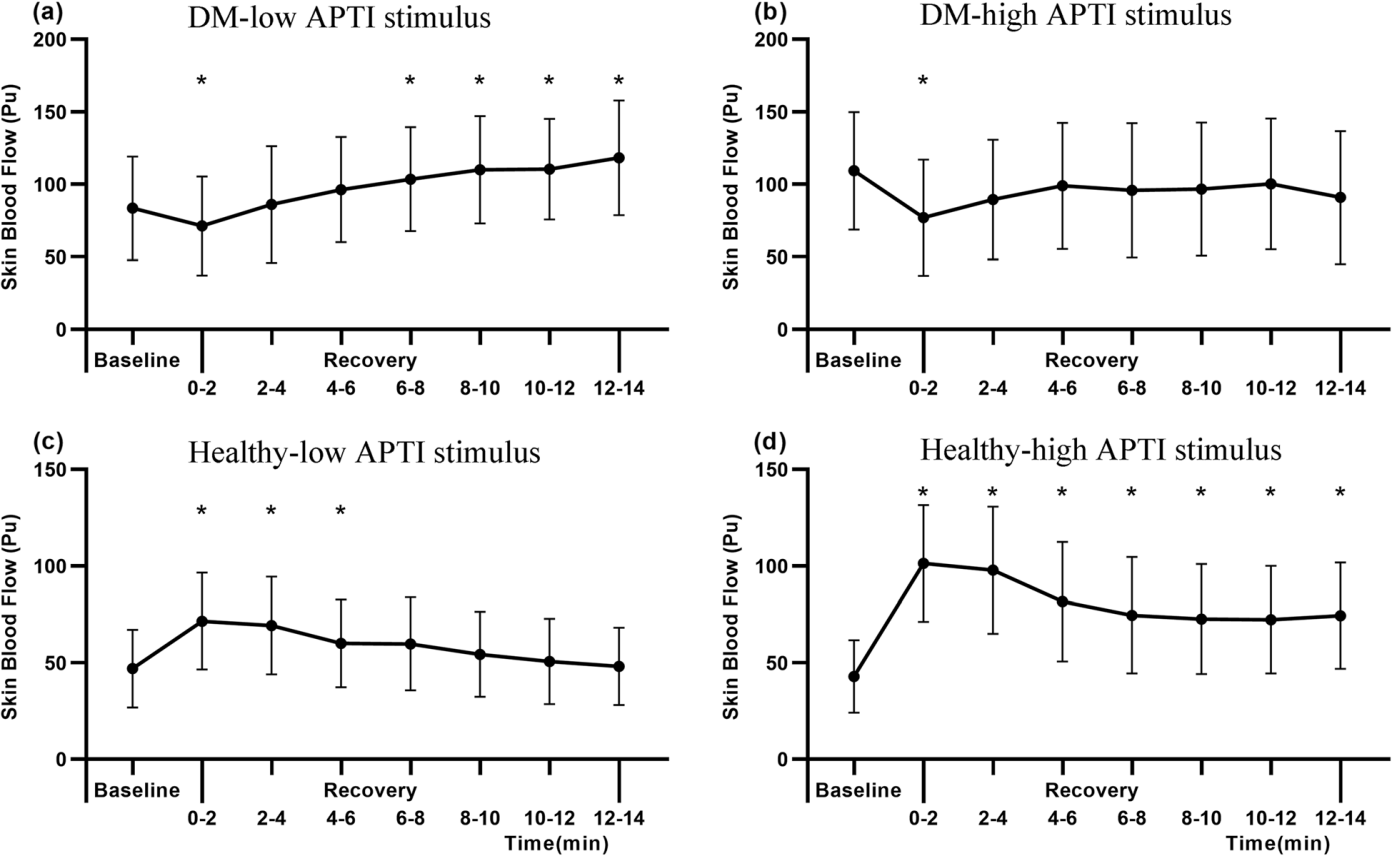
Results of skin blood flow during recovery and baseline in each group (mean with 95%CI). **a** Skin blood flow of DM group after the low APTI stimulus; **b** Skin blood flow of DM group after the high APTI stimulus; **c** Skin blood flow of healthy group after the low APTI stimulus; **d** Skin blood flow of healthy group after the high APTI stimulus. The symbol “*” indicates a significant difference between this period during recovery and baseline periods (*P* < 0.05)

Compared to plantar SBF during the baseline period, both SBF values significantly decreased in the first 2 min of the recovery period after both APTI stimuli in the DM group (low-APTI: 83.54 ± 49.89 vs 71.38 ± 47.65, high-APTI: 109.35 ± 52.74 vs 77.02 ± 52.08, *P* < 0.05), and then gradually recovered after 2 min. The SBF after 6 min in the recovery period was significantly increased under the low APTI stimulus compared baseline period (103.59 ± 50.16 vs 83.54 ± 49.89, *P* < 0.05), while the SBF after 2 min in the recovery period showed no significant difference from the baseline period under the high APTI stimulus (*P* > 0.05). However, compared to the plantar SBF during the baseline period, the healthy group showed a significant increase in SBF within 2 min after both APTI stimuli (low-APTI: 46.88 ± 42.95 vs 71.49 ± 53.35, *P* < 0.05; high-APTI: 42.84 ± 40.16 vs 101.36 ± 64.53, *P* < 0.05), which lasted until the 6th minute under the low APTI stimulus and the 14th minute under the high APTI stimulus (Fig. 3).

Within the DM group, people with DM were divided into a high tissue stiffness group (tissue stiffness: 33.36 ± 4.7 °shore) and a low tissue stiffness group (tissue stiffness: 19.4 ± 6.26 °shore) based on the mean value of the plantar tissue stiffness in DM group (26.38 ± 9.02 °shore). Normalized SBF of the high tissue stiffness group was lower than the low tissue stiffness group under the high APTI stimulus (0.66 ± 0.26 vs 1.12 ± 0.2; *P* < 0.05). However, no significant difference was observed in SBF between the high tissue stiffness group and the low tissue stiffness group under the low APTI stimulus (1.31 ± 0.53 vs 1.25 ± 0.17; *P* = 0.82).

## Discussion

This study compared the differences of plantar SBF responses after different APTI stimuli, including high APTI stimulus and low APTI stimulus in people with DM and healthy participants without DM. The results showed that normalized SBF of people with DM was significantly lower than that of healthy participants after the high APTI stimulus but not after the low APTI stimulus, which indicates that people with DM may have an impaired blood flow response under the high APTI stimulus. This study firstly demonstrated that plantar tissue showed different skin blood response under different accumulated loading stimuli even at the same walking speed. It indicates that the degree of microvascular dysfunction in people with DM may also be strongly associated with the magnitude of mechanical stress.

People with DM had a significantly lower skin blood flow response only under the high APTI stimulus in this study, which suggested that they may have a limited resilience to accumulated mechanical loading stimulus. Decreased blood flow has been considered to be associated with a higher risk of DFU, therefore, the findings of this study suggests that the accumulated mechanical stimulus could also be considered in the design or prescription of therapeutic shoe. Petrofsky et al. also found that people with DM have an impaired blood flow response under the high local transient (30s) pressure stimulus at the 2nd metatarsal head [23]. However, the pressure stimulus on the plantar tissue was applied by the fixed accumulated mechanical loading through a period of walking (the most common form of daily activities). The APTI at 73,000 × 1.5 kPa·s is approximately equal to the amount of pressure stimulus when people with DM walk for a little more than 20 min (PTI: 90 kPa·s [32]; step count: 54 steps per minute [31]). Although the American Diabetes Association (ADA) recommends that a bout of aerobic exercise should be maintained for at least 10 min [36], the prolonged abnormal pressure stimulus caused by high-intensity exercise is considered to have an adverse effect on the health of the plantar tissue [5, 15, 37]. Diminished skin blood flow of plantar tissue under the prolonged repetitive pressure stimulus may induce gangrene and increase the risk of DFUs. The findings of this study suggest that the diabetic plantar tissue is subjected to lower blood flow supply during a single continuous weight-bearing exercise with high APTI stimulus (e.g. longer walking duration or more walking steps), which may cause tissue inflammation and tissue damage, and thus accelerate the development of DFUs. Some studies support our finding and also indicate that multiple short-term exercise is preferred than a single long-term continuous exercise [38, 39]. However, Active exercise is proven to have greater health benefits for people with DM [12, 40]. ADA also recommended that people with DM should aim for 30 min/day or more of exercise and a cumulative exercise plan of 150 min per week [36]. Therefore, it may be crucial for people with DM to choose the appreciate type of exercise, such as continuous time and interval time of exercise. Future study should focus more on the effect of accumulated pressure stimulus caused by exercise on diabetic foot tissue to develop safer exercise plans.

This study also found that plantar SBF in people with DM decreased significantly within 2 min after both APTI stimuli, and then plantar SBF gradually increased. After the low APTI stimulus, the plantar SBF in people with DM was significantly higher than that in the baseline period from the 6th minute. However, the plantar SBF increased significantly within 2 min in healthy participants after both APTI stimuli. It indicates that people with DM may have a slowed blood flow response after walking. MacAnaney et al. also found that during the muscle relaxation period in a 6-min calf exercise, the responses of leg vascular conductance and muscle blood flow were profoundly slowed in women with DM. They indicated that this slowed hyperaemia reaction may be related to the impairment of endothelium-mediated vasodilation [41], which plays an important role in mediating smooth muscle relaxation [42, 43]. The external mechanical stimulus caused by daily walking would decrease plantar blood flow. When the pressure is released, smooth muscle cells and endothelial cells regulate vasodilation through electrical signals and vasodilator factors to increase skin blood flow [43–45]. Therefore, the slowed blood flow response after walking in people with DM may be because of impaired vasodilatory function, which failed to provide timely and sufficient blood flow to plantar tissue [46–48]. Pu et al. also demonstrated that people with DM no matter their PPP levels showed a similar decrease in SBF within 2 min after 73,000 kPa·s walking stimulus (i.e. the low APTI stimulus in this study) [18]. It suggests that people with DM should take an appropriate rest time (e.g. at least 2 min) after walking to ensure the recovery of plantar skin blood flow.

In this study, the walking speed was controlled at 3.2 km/h to apply the preset value of ATPI to the plantar foot. The results showed that normalized SBF of healthy participants increased significantly after the high APTI stimulus. Wu et al. demonstrated that faster walking speeds could significantly increase plantar skin blood flow within the same duration, and Burnfield et al. demonstrated that the faster walking speed increased the peak plantar pressure [14]. That is, the plantar tissue may be subjected to different accumulated pressure stimulus when walking at different speeds for the same duration. Therefore, the effect of walking speed on the plantar blood flow responses found by Wu et al. may have a relationship with the different APTI stimuli applied to the plantar tissue. It suggests that APTI may also play an important role in evaluating the effects of daily walking and other weight-bearing exercises on the plantar microvascular.

This study also found that normalized SBF of the high tissue stiffness group was significantly lower than the low tissue stiffness group in people with DM under the high APTI stimulus. Jan et al. demonstrated that tissue stiffness is an important factor affecting skin blood flow response to mechanical stress [28]. Increased plantar tissue stiffness in people with DM decreases the ability to effectively distribute the pressure during weight-bearing exercise, which is more likely to cause skin breakdown and develop foot ulcers [27, 49, 50]. Previous study has demonstrated that the increase of skin stiffness in people with diabetic neuropathy strongly correlates with the severity of neuropathy [27, 51]. Periyasamy et al. ‘s study reported that the tissue stiffness in the first metatarsal area of people with diabetic neuropathy and people without DM was 42.8 ± 12.0 °shore and 28.2 ± 4.1 °shore (mean ± SD), respectively [29]. In this study, none of participants with DM have peripheral neuropathy or foot deformity and the plantar tissue stiffness of the first metatarsal area was lower than 42.8 °shore, which means that people with DM in this study have not developed severe stiffening of plantar soft tissue. However, in this repeated measures experiments, people with high tissue stiffness still had a lower blood flow response compared to people with low tissue stiffness, which suggested that plantar tissue stiffening may have a negative effect on plantar blood flow of diabetic plantar tissue even without peripheral neuropathy. Therefore, for people with high plantar tissue stiffness, the accumulated pressure stimulus on the plantar foot during daily walking should be of greater concern in order to ensure sufficient blood flow of the plantar tissue.

Research studies have shown that both aging and diabetes are associated with impaired vasodilatory function [23, 52]. It was also demonstrated that the accumulation of advanced glycation end-products (AGEs) in tissues increases with age [53], which may accelerate the production of oxidative stress in the cells, thereby causing an inflammatory response [54]. A global epidemiology study of DFUs [3] has shown that people with DFUs are older age compared to people without DFUs (61.7 ± 3.7 vs 56.1 ± 3.9 years), which indicated that the older age may also be associated with a higher risk of DFUs. These findings suggest that elderly people with DM may have more serious microvascular damage than young people with DM. Young healthy people may contributed to establish the normal response of plantar tissue to various accumulated pressure-time integral stimuli. For people with diabetes, decreased plantar blood flow under high APTI stimulus may have a negative effect on the supply of nutrient and oxygen to plantar tissue, thus aggravating the plantar tissue viability and increasing risk for DFUs. Although the impaired blood flow reactivity in people with DM (age: 68.1 ± 4.61 years) observed in this study may be affected by both aging and diabetes, the results of this study can provide some guidelines for older people with DM to perform safe exercise training.

There are limitations in this study. Firstly, this study only compared two intensities of APTI stimulus. Higher APTI stimulus means that the plantar tissue is under a larger physiologic stress that requires a longer time to overcome this stress by removing metabolic wastes and restoring oxygenation of the affected tissue [19, 42]. It may have a negative impact on the viability of diabetic foot tissue. In the future, different levels of APTI stimuli need to be investigated to determine the safe accumulated pressure-time threshold. Secondly, this study only focused on the area with high incidence of DFUs. Future studies may focus on other areas, such as heels or toes, which may also be at risk for diabetic foot ulcers [55]. Thirdly, this study cannot discuss the effect of tissue stiffness on plantar skin blood flow in people with DM in detail due to the small sample size and the absence of tissue stiffness in healthy people. It is necessary to carry out more experiments to explore the relationship between tissue stiffness and the risk of diabetic foot ulcers. Fourthly, people with DM in this study were older due to a greater risk of DFUs in older people. The results may not be generalized to young people with DM. Future research may need to investigate the plantar blood flow response of older people without diabetes and young people with diabetes under different APTI stimuli to further investigate the role of APTI on the development of DFUs. Last, we used laser Doppler flowmetry to measure plantar SBF for assessing plantar tissue viability after various intensities of plantar tissue stress in this study. Future research may need to conduct a longitudinal study to evaluate whether high plantar stresses of weight-bearing physical activities are associated with higher incidence of DFU in people with diabetic peripheral neuropathy.

## Conclusions

In conclusion, this study demonstrated that people with DM had a normal plantar blood flow response after walking at the low APTI stress and had an impaired blood flow response after walking at the high APTI stress. This indicates a need to establish a safe range plantar tissue stress of weight-bearing physical activity in people at risk for DFUs. The results indicated that the accumulated mechanical stimulus could also be considered in the design or prescription of therapeutic shoes to avoid hypoperfusion under prolonged weight-bearing exercise with high APTI stimulus. Our study also demonstrated that people with DM should take a rest for more than 2 min to allow a full vasodilatory response to restore plantar skin blood flow after weight-bearing exercise. These findings may provide guidance for clinicians in prescribing weight-bearing exercise regimens for people with DM.

## Data Availability

All data generated or analysed during this study are included in this published article.

## Abbreviations

APTI: Accumulated pressure-time integral
SBF: Skin blood flow
DM: Diabetes mellitus
DFUs: Diabetic foot ulcers
PPP: Peak plantar pressure
LDF: Laser Doppler Flowmetry
BMI: Body mass index
ABI: Ankle brachial index.
SBP: Systolic blood pressure
DBP: Diastolic blood pressure
ADA: American Diabetes Association
AGEs: Advanced glycation end-products
